# *Spirulina platensis* Inclusion Reverses Circulating Pro-inflammatory (Chemo)cytokine Profiles in Broilers Fed Low-Protein Diets

**DOI:** 10.3389/fvets.2021.640968

**Published:** 2021-05-10

**Authors:** Garrett J. Mullenix, Elizabeth S. Greene, Nima K. Emami, Guillermo Tellez-Isaias, Walter G. Bottje, Gisela F. Erf, Michael T. Kidd, Sami Dridi

**Affiliations:** Center of Excellence for Poultry Science, University of Arkansas, Fayetteville, AR, United States

**Keywords:** broilers, *Spirulina*, low protein diet, inflammation, cytokines, chemokines, inflammasome

## Abstract

Proteins are considered the most expensive nutrients in commercial modern broiler production, and their dietary inclusion at low levels is pivotal to minimize feed costs and reduce nitrogen waste. The quest for an environmentally friendly source of proteins that favor the formulation of low protein diets without compromising broiler health, welfare, and growth performance has become a hotspot in nutrition research. Due to its high protein content, the naturally growing *Spirulina* microalgae is considered a promising nutrient source. The purpose of the present study was, therefore, to determine the effects of *Spirulina* supplementation on liver bacterial translocation, hematological profile, and circulating inflammatory and redox markers in broilers fed a low-protein diet. One-day-old Ross 708 male broilers (*n* = 180) were randomly assigned into one of three experimental treatments: standard diet as a control, low protein diet, and low protein diet supplemented with 100 g/kg of *Spirulina*. Target molecular markers were measured in the peripheral blood circulation using real-time quantitative PCR. Reducing dietary proteins increased bacterial translocation and systemic inflammation as indicated by proportions of basophils among blood leukocytes. The expression levels of circulating pro-inflammatory cytokines [interleukin (IL)-3, IL-6, IL-4, IL-18, and tumor necrosis factor-α], chemokines (CCL-20), and NOD-like receptor family pyrin domain containing 3 inflammasome were significantly upregulated in birds fed the low protein diet compared with the control. The inclusion of *Spirulina* reversed these effects, which indicates that *Spirulina* reduces systemic inflammation- and bacterial translocation-induced by a low protein diet and could be a promising alternative protein source in poultry diets.

## Introduction

Global poultry meat production is projected to increase by 32% by 2030 and 59% by 2050 from production in 2012, with low- and middle-income countries making up most of this demand (1). The cost of feed, particularly proteins, and availability of feedstuffs are by far the most important factors in poultry production sustainability and competitiveness. Indeed, soybean meal (SBM) is the preferred source of protein in broiler diets, as it is the only economically feasible protein source currently used in many countries. However, the continuous increase in global demand for high-quality animal protein, which consequently needs an improved animal production and abundant animal feeds, may inevitably surpass the SBM supply. This may, in turn, affect the inflation and the market price of SBM, which is linearly rising. Furthermore, there has also been a precedent set to limit animal-derived protein sources for animal feed in the European Union *via* Regulation (EC) No. 999/2001 (2). Thus, numerous innovative and effective approaches will need to be implemented to breach this looming protein gap to keep up with the worldwide demand for poultry meat.

Reducing crude protein (CP) in broiler diets has been extensively studied, yet the underlying issues of increased fat deposition and decreased performance are still prevalent (3–5). Implementing the ideal protein concept, computer least-cost formulation, and commercially produced crystalline amino acids allow for the lowest dietary CP diet thus far (6, 7). The reduction of CP with supplemental crystalline amino acids alleviates the demand for SBM, yet alternative protein sources will also be important for meeting the anticipated increase in demand for protein in animal and poultry feed. Alternatives for SBM in broiler diets have been investigated, including traditional and novel options such as canola meal, blood meal, meat and bone meal, fish meal, peameal, sunflower meal, insect meal, and numerous algae species (8–12). Many of the trials involving algae in broiler feed have focused on growth performance or meat quality with limited mechanistic explanations for their beneficial effects. Algae inclusion rates of up to 100 g/kg have maintained or increased performance parameters in chickens reared under conventional conditions (13–15).

*Spirulina* (*Athrospira sp*.) *platensis* is a filamentous spiral-shaped blue-green cyanobacterium that grows naturally in warm and alkaline aquatic media and is particularly interesting as a possible animal feed protein source due to its high level of CP (43–70%) and balanced amino acid profile. In addition to essential fatty acids, vitamins, minerals, and pigments (16), *Spirulina* contains several compounds shown to have antioxidant, anti-inflammatory, immune-modulating, and probiotic properties (17–20). *Spirulina* is also considered a nutritionally safe feed ingredient with no risk of mineral toxicity and free of the major algal toxin, microcystins (21, 22). *Spirulina* use as a feed ingredient for multiple species has been outlined (23), and one of the confounding issues put forth is the underlying mechanisms by which *Spirulina* impacts them. The purpose of this study was, therefore, to determine hematological parameters, inflammatory markers, and oxidative stress in broilers fed a low CP diet with and without *Spirulina*.

## Materials and Methods

### Animals and Treatment

A total of 180 1-day-old male (Ross 708) broiler chicks were obtained from a commercial hatchery and randomly allotted to one of the 15-floor pens (5 pens/diet; 12 birds/pen) in an environmentally controlled pilothouse. Chicks were reared in pens top-dressed with ~4 cm of fresh wood shavings, and the temperature gradually decreased from 32°C on day 1 to 20°C on day 27. The trial was conducted from February to March 2020. Birds received 23 h of light until day 10; then, light duration was decreased to 18 h for the remainder of the trial. Birds were given *ad libitum* access to feed (Table 1) and water throughout the trial. A standard corn–SBM basal diet (3,250 kcal/kg^−1^, 21% CP) was fed to all birds until day 14, at which point experimental diets were introduced until 37 days of age. The experimental diets included an industry-standard level protein (~20% CP) corn/SBM control (SCP), reduced (~17%) CP corn/SBM diet (LCP), and LCP diet where *Spirulina* was included at the level of 100 g/kg (SP-LCP). All experimental diets were isocaloric and met all essential amino acid needs set forth by the primary breeder. Both low CP diets were formulated to be isonitrogenous.

**Table 1:** Composition of experimental diets.

**Parameter**	**SCP**	**LCP**	**SP-LCP**
Ingredient (g/kg)			
Maize	633.1	718.3	745.9
Soybean meal (48%)	312.3	221.6	104.5
*Spirulina* Algae	0.0	0.0	100.0
Poultry fat	21.8	14.5	9.3
Dicalcium phosphate	7.79	8.4	4.4
Limestone	10.8	11.1	13.9
Salt	3.7	1.0	0.5
Sodium bicarbonate	1.4	5.8	5.8
Choline-Cl	0.8	1.1	1.8
Mineral Premix^*^	1.0	1.0	1.0
Vitamin Premix^*^	0.4	0.4	0.4
Phytase	0.1	0.1	0.1
Coccidiostat	0.5	0.5	0.5
DL-Methionine	3.1	3.8	2.7
L-Lysine HCl	2.1	4.9	5.1
L-Threonine	1.1	2.3	1.2
L-Arginine	0.0	2.2	2.3
L-Valine	0.0	1.59	0.3
L-Isoleucine	0.0	1.29	0.3
L-Tryptophan	0.0	0.2	0.2
Estimated compostion (g/kg)			
Crude protein	201	170	170
AME (MJ/kg)	14.1	14.3	14.3
Calcium	8.4	8.4	8.4
Available phos.	4.2	4.2	4.2
Met + Cys	9.1	9.0	9.4
Lys	12.0	11.8	12.1
Thr	8.5	8.3	8.5
Ile	8.4	7.9	8.4
Val	9.3	9.1	9.6
Trp	2.4	2.0	2.1
Arg	13.0	12.3	13.0
Digestible Met + Cys	8.4	8.4	8.4
Digestible Lys	11.0	11.0	11.0
Digestible Thr	7.4	7.4	7.4
Digestible Ile	7.5	7.3	7.3
Digestible Val	8.3	8.3	8.3
Digestible Trp	2.1	1.8	1.8
Digestible Arg	12.1	11.6	11.6
L-Threonine	1.1	2.3	1.2

* *Added per kg of finished feed: Vitamin A, 30,864 IU; vitamin D3, 22,046 IU; vitamin E, 220 IU; vitamin B12, 0.05 mg; menadione, 6.0 mg; riboflavin, 26.5 mg; D-pantothenie acid, 39.7; thiamine, 6.2 mg; niacin, 154.3; pyridoxine, 11.0 mg; folic acid, 3.5 mg; biotin, 0.33 mg; zinc, 100; iron, 15; manganese, 100; copper, 15; iodide, 1.20; selenium, 0.25; calcium, 69*.

### Blood Sampling and Hematological Analysis

On day 37, blood (3 ml) was collected from non-fasted birds (10 birds/treatment; 2 birds/pen), *via* the brachial wing vein, in ethylenediaminetetraacetic acid-coated tubes and immediately placed on ice. The hematologic profile of 1 ml of whole blood samples was measured using the Cell-Dyn 3500 automated hematology analyzer calibrated for chicken blood (Abbott Diagnostics, Abbott Park, IL) within 3 h of sampling. Data collected included the percent of heterophils, lymphocytes, monocytes, eosinophils, and basophils and the calculated heterophil to lymphocyte (H/L) ratio. For gene expression analysis, 250 μl of blood was added to tubes containing 750 μl of TRIzol LS reagent according to manufacturer's recommendations (Life Technologies Corporation, CA, US). The birds' bodyweights were 2.61 ± 0.07, 2.60 ± 0.07, and 2.72 ± 0.08 kg for SCP, LCP, and SP-LCP, respectively.

### Liver Bacterial Translocation

As previously described by Tellez et al. (24), a section of the right liver was aseptically removed from 30 chickens (10 birds/treatment), placed into sterile sampling containers, and homogenized. Samples were then diluted 1:4 based on tissue weight with sterile 0.9% saline. Liver samples were then transferred to sterile 96-well Bacti flat-bottom culture plates and diluted 10-fold before being plated on tryptic soy agar to evaluate total counts of *Enterobacteriaceae* per gram of tissue. Samples were incubated under aerobic conditions at 37°C for 24 h.

### RNA Isolation, Reverse Transcription, and Quantitative Real-Time PCR

Total RNA was isolated from whole blood samples using Trizol LS reagent (ThermoFisher Scientific, Rockford, IL) according to manufacturer's recommendations. Take 3 Micro-Volume Plate using Synergy HT multimode microplate reader (BioTek, Winooski, VT) determined RNA concentrations and purity. Real-time quantitative PCR (Applied Biosystems 7500 Real-Time PCR System) was performed using 5 μl of complementary DNA, 1 μl of each forward and reverse specific primers, and 10 μl of SYBR Green Master Mix (ThermoFisher Scientific, Rockford, IL, United States) in a total 25-μl reaction. Oligonucleotide primers used for chicken cytokines, chemokines, inflammasomes, anti-oxidative, and 18S (housekeeping) genes are summarized in Table 2. The real-time quantitative PCR cycling conditions were 50°C for 2 min, 95°C for 10 min followed by 40 cycles of a two-step amplification program (95°C for 15 s and 58°C for 1 min). At the end of the amplification, melting curve analysis was applied using the dissociation protocol from the Sequence Detection system to exclude contamination with unspecific PCR products. 18S RNA was used to normalize the relative expression of targeted genes *via* the 2^−ΔΔCt^ method (25). All values were compared relative with those in the SCP dietary control group.

**Table 2:** Oligonucleotide real-time qPCR primers.

**Gene**	**Accession number^**a**^**	**Primer sequence (5′ → 3′)**	**Orientation**	**Product size (bp)**
TNFα	NM_204267	CGTTTGGGAGTGGGCTTTAA	Forward	61
		GCTGATGGCAGAGGCAGAA	Reverse	
IL-18	GU119895	TGCAGCTCCAAGGCTTTTAAG	Forward	63
		CTCAAAGGCCAAGAACATTCCT	Reverse	
IL-3	NM_001007083.1	CAGCACCTCCTCCCTGTCA	Forward	64
		GGCTTCATTGCTGCCCTGTA	Reverse	
IL-4	NM_0010079.1	GCTCTCAGTGCCGCTGATG	Forward	60
		GAAACCTCTCCCTGGATGTCAT	Reverse	
IL-10	NM_001004414.2	CGCTGTCACCGCTTCTTCA	Forward	63
		CGTCTCCTTGATCTGCTTGATG	Reverse	
IL-6	NM_204628.1	GCTTCGACGAGGAGAAATGC	Forward	63
		GGTAGGTCTGAAAGGCGAACAG	Reverse	
C3	NM_205405.3	CCAGAGCCTGGTCACGATGT	Forward	62
		CGATACGGAAGGAAGGGATGA	Reverse	
CRP	NM_001039564	AAGCTCAGGACAACGAGATCCT	Forward	71
		TTTCCCCCCCACGTAGAAG	Reverse	
NLRP3	XM_001233261	GTTGGGCAGTTTCACAGGAATAG	Forward	63
		GCCGCCTGGTCATACAGTGT	Reverse	
NLRC3	XM_015294675.2	CTCCAACGCCTCACAAACCT	Forward	93
		GCCTTTGGTCATTTCCATCTG	Reverse	
NLRC5	NM_001318435.1	CTCGAAGTAGCCCAGCACATT	Forward	80
		CATGTCCAGAGGTGTCAGTCTGA	Reverse	
NLRX1	XM_003642592.4	GGCTGAAACGTGGCACAAA	Forward	59
		GAGTCCAAGCCCAGAAGACAAG	Reverse	
GPx1	NM_001277853.2	TCCCCTGCAACCAATTCG	Forward	57
		AGCGCAGGATCTCCTCGTT	Reverse	
GPx3	NM_001163232.2	GGGCGCTGACCATCGAT	Forward	59
		CATCTTCCCCGCGTACTTTC	Reverse	
SOD1	NM_205064.1	TGGCTTCCATGTGCATGAAT	Forward	58
		AGCACCTGCGCTGGTACAC	Reverse	
SOD2	NM_204211.1	GCTGGAGCCCCACATCAGT	Forward	61
		GGTGGCGTGGTGTTTGCT	Reverse	
TLR-3	NM_001011691.3	GATTGCACCTGTGAAAGCATTG	Forward	67
		CGGGTATATATGCTTGAGTGTCGTT	Reverse	
TLR-4	NM_001030693.1	TCCTCCAGGCAGCTATCAAGAT	Forward	74
		GACAACCACAGAGCTCATGCA	Reverse	
TLR-21	NM_001030558.1	CTGCTGACCGACCTCTATCACA	Forward	61
		GGTTGAGGGTGCGCAGTCT	Reverse	
MyD88	NM_001030962.4	ACCTCAAAGATCATCCAGTCCAA	Forward	63
		TGGGACACAGTCAGTGTGCAT	Reverse	
CCLL-4	NM_001045831.1	CTTGCTGTCGGGTCCAATG	Forward	60
		CGAGGGAAGTGCTCTGTTTAAGA	Reverse	
CXCL-14	NM_204712.2	CCGGCTCGCCATGAAG	Forward	54
		ATCGCGATGACCAGCAGAA	Reverse	
CCL-4	NM_204720.1	CCTGCTGCACCACTTACATAACA	Forward	63
		TGCTGTAGTGCCTCTGGATGA	Reverse	
CCL-20	NM_204438.2	TGCTGCTTGGAGTGAAAATGC	Forward	62
		CAGCAGAGAAGCCAAAATCAAA	Reverse	
18s	AF173612	TCCCCTCCCGTTACTTGGAT	Forward	60
		GCGCTCGTCGGCATGTA	Reverse	

a *Accession number refer to Genbank (National Center for Biotechnology Information). TNF-α, tumor necrosis factor-alpha; IL, interleukin; C3, complement component 3; CRP, C-reactive protein; NLR, NOD-like receptor; GPX, glutathione peroxidase; SOD, superoxide dismutase; TLR, toll-like receptor; MyD88, myeloid differentiation primary response 88; CCLL4, chemokine-like ligand; CXCL, C-X-C motif chemokine ligand; CCL, C-C motif chemokine ligand*.

### Statistical Analysis

All data were analyzed as a complete randomized design with one-way ANOVA in JMP Pro v 15.0 (SAS Institute, Cary, NC, United States). *Post-hoc* analysis assessment through multiple Dunnett comparisons was used when appropriate. Differences were considered significant at *P* ≤ 0.05.

## Results

### Hematological Analysis

Reducing CP by 3% in the diet (LCP) increased lymphocyte percent (*P* < 0.01) while decreasing that of heterophils (*P* = 0.026) and thereby resulted in a significantly lower H/L ratio (*P* = 0.014) (Table 3). The LCP group exhibited a significantly higher percent of basophil but no differences in monocyte or eosinophil percent compared with the SCP group. The inclusion of *Spirulina* reversed basophil percent to a similar level as the SCP (control) group. *Spirulina* inclusion had no impact on the H/L ratio or the percent of heterophils, lymphocytes, monocytes, or eosinophils (Table 3).

**Table 3:** Effects of a standard corn/soy (SCP), low crude protein (LCP), and *Spirulina* included low crude protein diet (SP-LCP) on blood lymphocyte profiles of broilers on d 37.

**Parameter**	**SCP**	**LCP**	**SP-LCP**	**SEM**	***P-*value**
Heterophils, %	55.21^a^	41.34^b^	41.80^b^	2.975	0.010
Lymphocytes, %	39.14^b^	48.39^a^	52.11^a^	2.982	0.026
Heterophil/lymphocyte	1.47^a^	0.86^b^	0.84^b^	0.143	0.014
Monocytes, %	2.16	3.00	2.62	0.467	0.472
Eosinophils, %	0.12	0.14	0.14	0.036	0.901
Basophils, %	3.37^b^	7.13^a^	3.33^b^	0.854	0.012

a, b *means in each row with different superscripts are significantly different (P < 0.05)*.

### Liver Bacterial Translocation

The presence of bacteria in the liver reflects bacterial translocation from the gastrointestinal tract. Bacterial counts in the liver were elevated (*P* < 0.05) by the LCP diet compared with birds fed with the SCP control diet (3.34 ± 0.7 vs. 2.44 ± 0.4 log_10_ CFU/g). Bacterial translocation was ameliorated in birds fed the *Spirulina* LCP diet to levels (1.34 ± 0.4 log_10_ cfu/g) lower than in the liver of birds fed the SCP control diet.

### Circulating Inflammation-, Toll-Like Receptor-, and Antioxidant-Associated Markers

**Figure 1:**
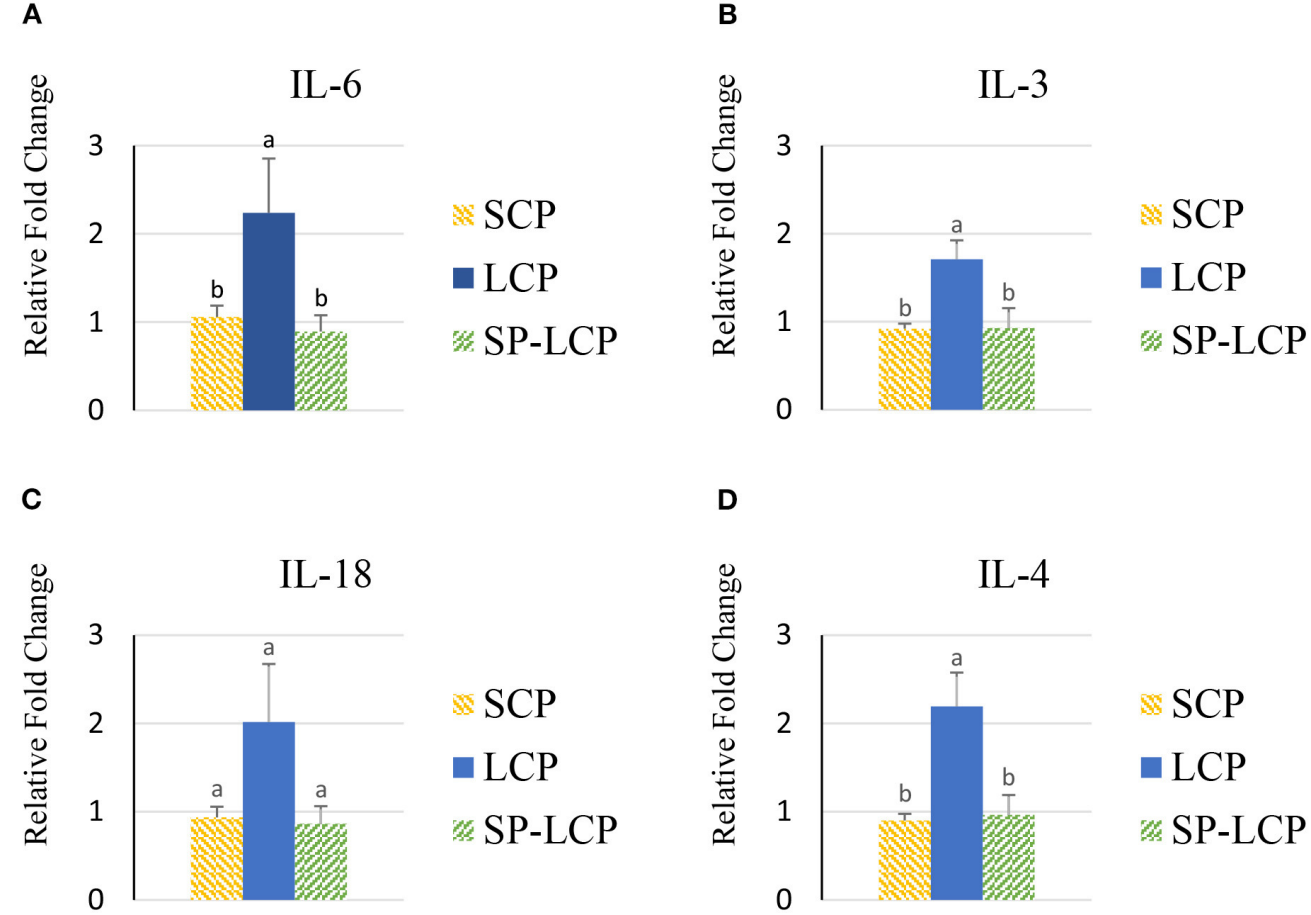
Effects of *Spirulina* on circulating cytokine expression profile. Relative mRNA abundance of circulating IL-6 **(A)**, IL-3 **(B)**, IL-18 **(C)**, and IL-4 **(D)** in broilers on day (d) 37 were determined by real-time qPCR. Treatments include standard corn/soy (SCP) diet as control and low crude protein without (LCP) or with *Spirulina* (SP-LCP). Data are presented as mean ± SEM (*n* = 8/group). Means with different letters are significantly different (*P* < 0.05).

**Figure 2:**
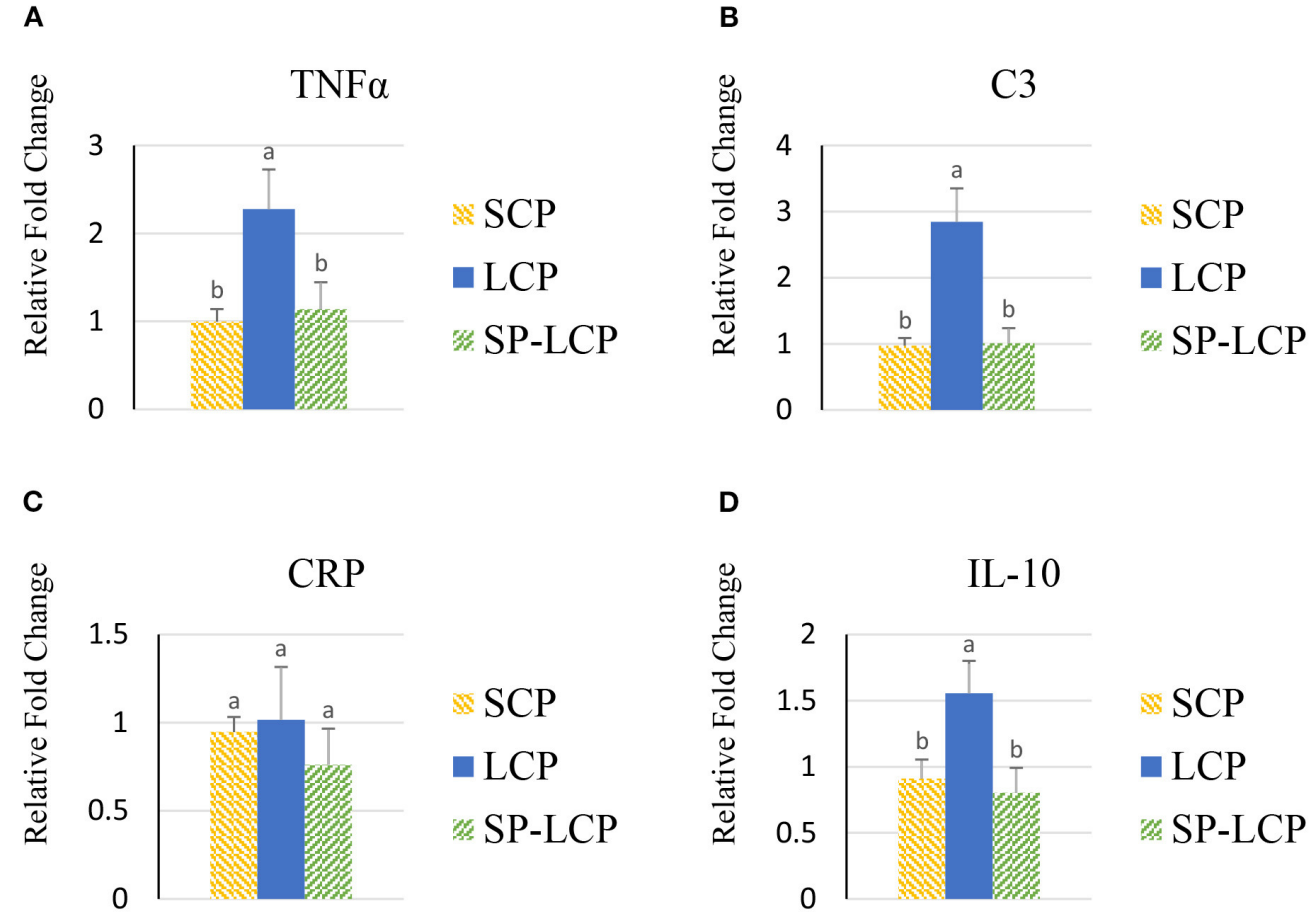
Effects of *Spirulina* on circulating TNFα, C3, CRP, and IL-10 expression. Relative mRNA abundance of circulating TNF-α **(A)**, C3 **(B)**, CRP **(C)**, and IL-10 **(D)** expression in broilers on day (d) 37 were determined by real-time qPCR. Treatments include standard corn/soy (SCP) diet as control and low crude protein without (LCP) or with *Spirulina* (SP-LCP). Data are presented as mean ± SEM (*n* = 8/group). Means with different letters are significantly different (*P* < 0.05).

**Figure 3:**
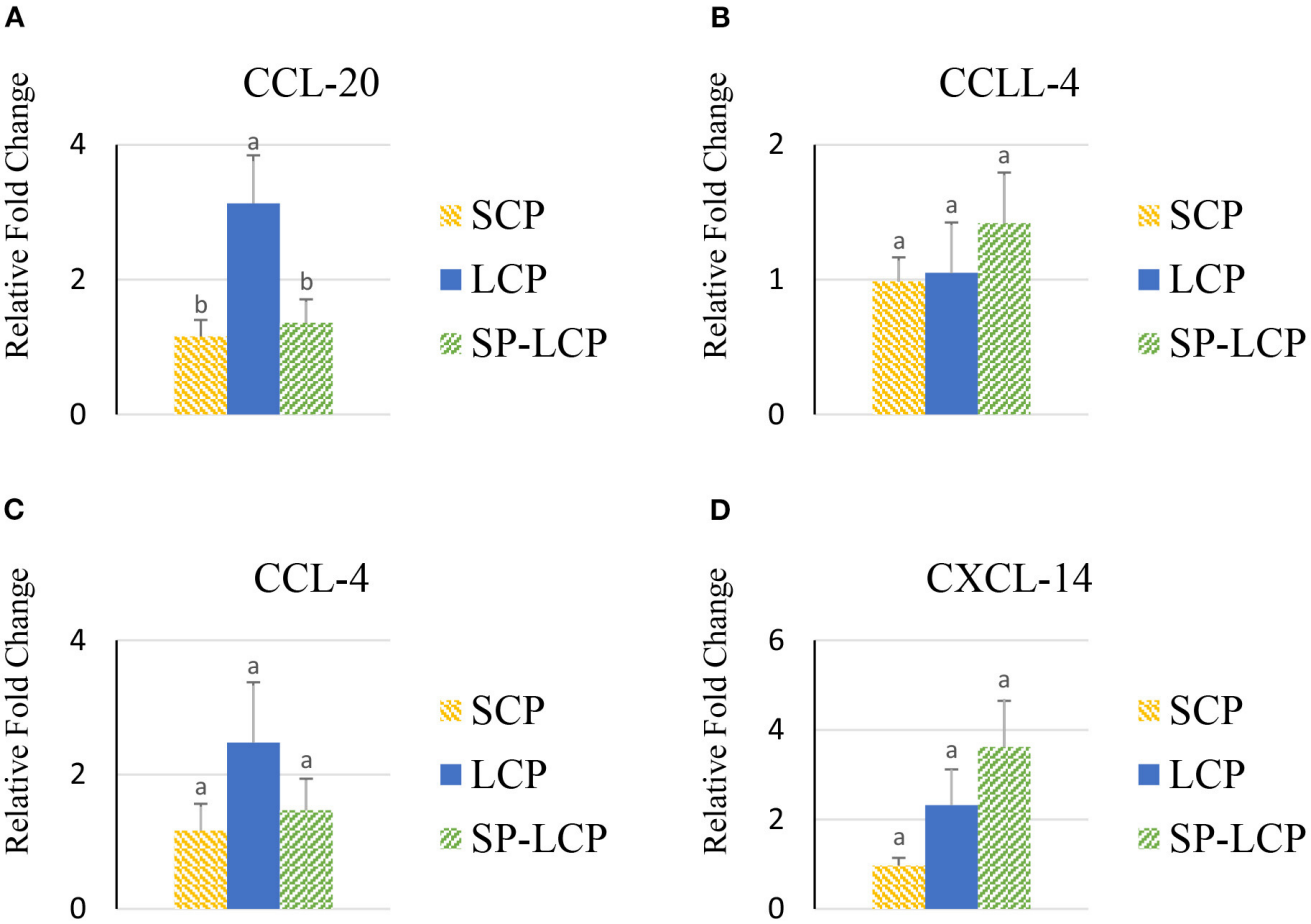
Effects of *Spirulina* on circulating chemokine expression profile. Relative mRNA abundance of circulating chemokine (C-C motif) ligand 20 (CCL-20) **(A)**, chemokine-like ligand 4 (CCLL-4) **(B)**, chemokine (C-C motif) ligand 4 (CCL-4) **(C)**, and chemokine (C-X-C motif) ligand 14 (CXCL-14) **(D)** in broilers on day (d) 37 were determined by real-time qPCR. Treatments include standard corn/soy (SCP) diet as control and low crude protein without (LCP) or with *Spirulina* (SP-LCP). Data are presented as mean ± SEM (*n* = 8/group). Means with different letters are significantly different (*P* < 0.05).

**Figure 4:**
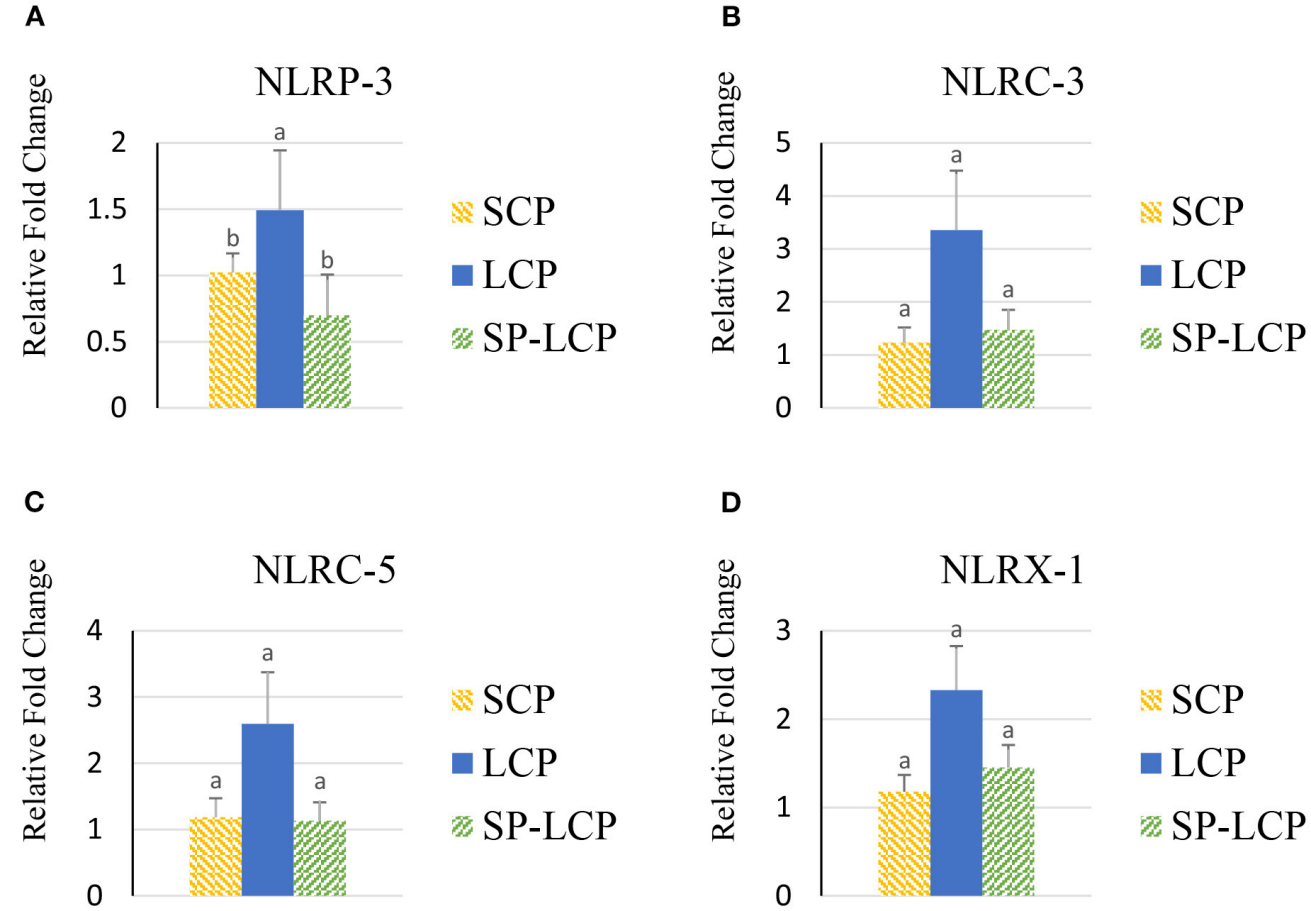
Effects of *Spirulina* on circulating inflammasome expression. Relative mRNA abundance of NOD-like receptor family pyrin domain-containing 3 (NLRP-3) **(A)**, NOD-like receptor family CARD domain-containing 3 (NLRC-3) **(B)**, NOD-like receptor family CARD domain-containing 5 (NLRC-5) **(C)**, and nucleotide-binding oligomerization domain, leucine-rich repeat-containing X1 (NLRX-1) **(D)** in broilers on day (d) 37 were determined by real-time qPCR. Treatments include standard corn/soy (SCP) diet as control and low crude protein without (LCP) or with *Spirulina* (SP-LCP). Data are presented as mean ± SEM (*n* = 8/group). Means with different letters are significantly different (*P* < 0.05).

**Figure 5:**
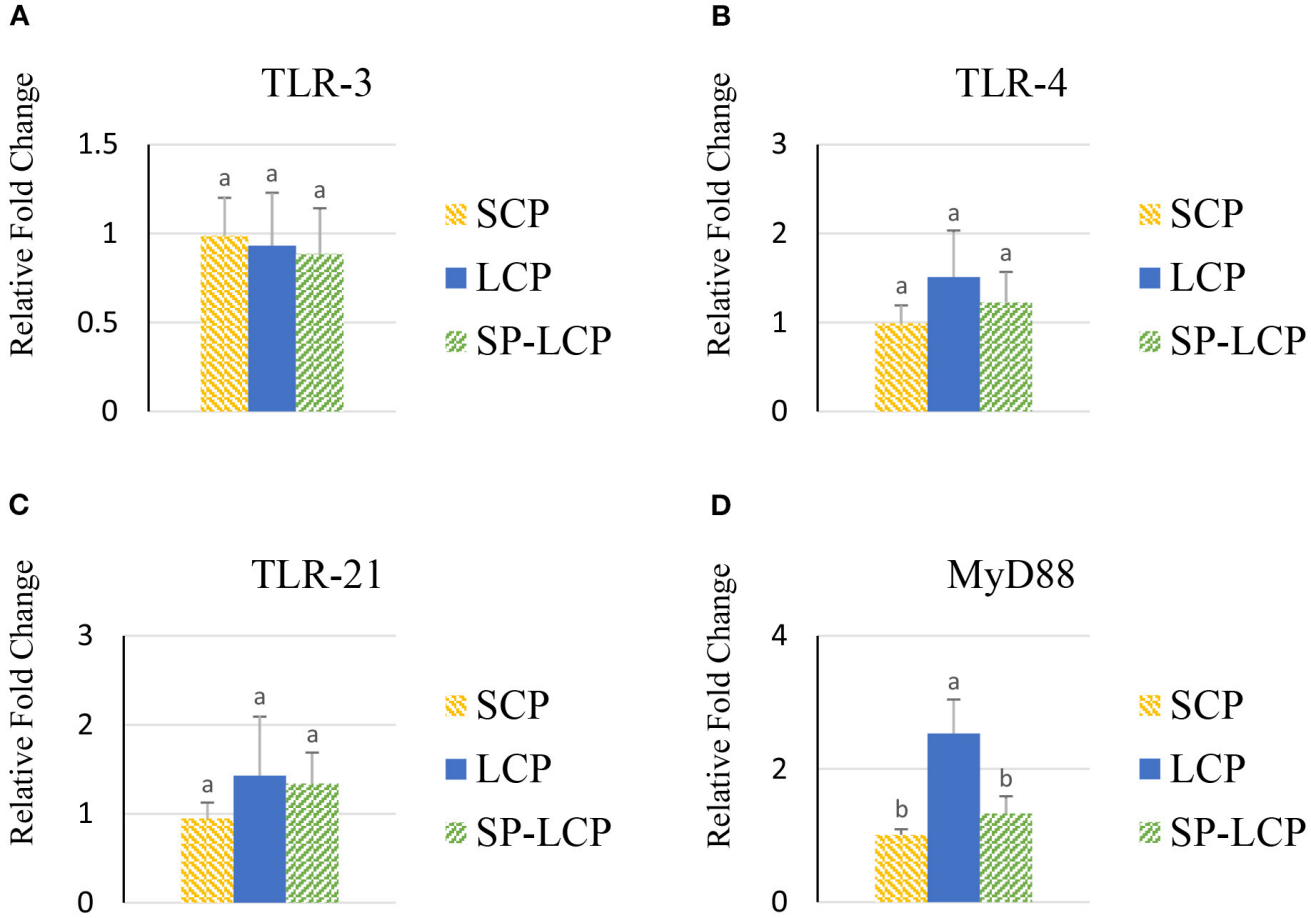
Effects of *Spirulina* on circulating toll-like receptor expression. Relative mRNA abundance of toll-like receptor 3 (TLR-3) **(A)**, toll-like receptor 4 (TLR-4) **(B)**, toll-like receptor 21 (TLR-21) **(C)**, and myeloid differentiation primary response protein 88 (MyD88) **(D)** in the blood of broilers on day (d) 37 were determined by real-time qPCR. Treatments include standard corn/soy (SCP) diet as control and low crude protein without (LCP) or with *Spirulina* (SP-LCP). Data are presented as mean ± SEM (*n* = 8/group). Means with different letters are significantly different (*P* < 0.05).

The LCP diet upregulated (*P* < 0.05) the expression of pro-inflammatory cytokines interleukin-6 (IL-6), interleukin-3 (IL-3), interleukin-18 (IL-18), and tumor necrosis factor-alpha (TNF-α), as indicated by expression of lipopolysaccharide-induced TNF-α factor (Figures 1A–D, 2A) and that of the regulatory cytokine interleukin-10 (IL-10) and interleukin-4 (IL-4) (Figure 2D). Similarly, the LCP diet upregulated the expression of circulating chemokine C-C motif ligand 20CCL-20 (Figure 3A) but not that of C-C motif ligand 4 or C-X-C motif ligand 14 (Figures 3B–D). LCP supplementation increased the messenger RNA (mRNA) abundance of NOD-like receptor family pyrin domain-containing 3 but not NOD-like receptor family CARD domain-containing 3, NOD-like receptor family CARD domain-containing 5, and nucleotide-binding oligomerization domain and leucine-rich repeat-containing X1 inflammasomes (Figures 4A–D). LCP diet upregulated the expression of myeloid differentiation primary response protein 88 (MyD88) but not that of toll-like receptors 3, 4, and 21 (TLR-3, TLR-4, TLR-21, and MyD88) (Figures 5A–D). *Spirulina* inclusion reversed the expression of all of the markers mentioned earlier to levels being expressed in birds fed with the SCP control diet (Figures 1–4).

**Figure 6:**
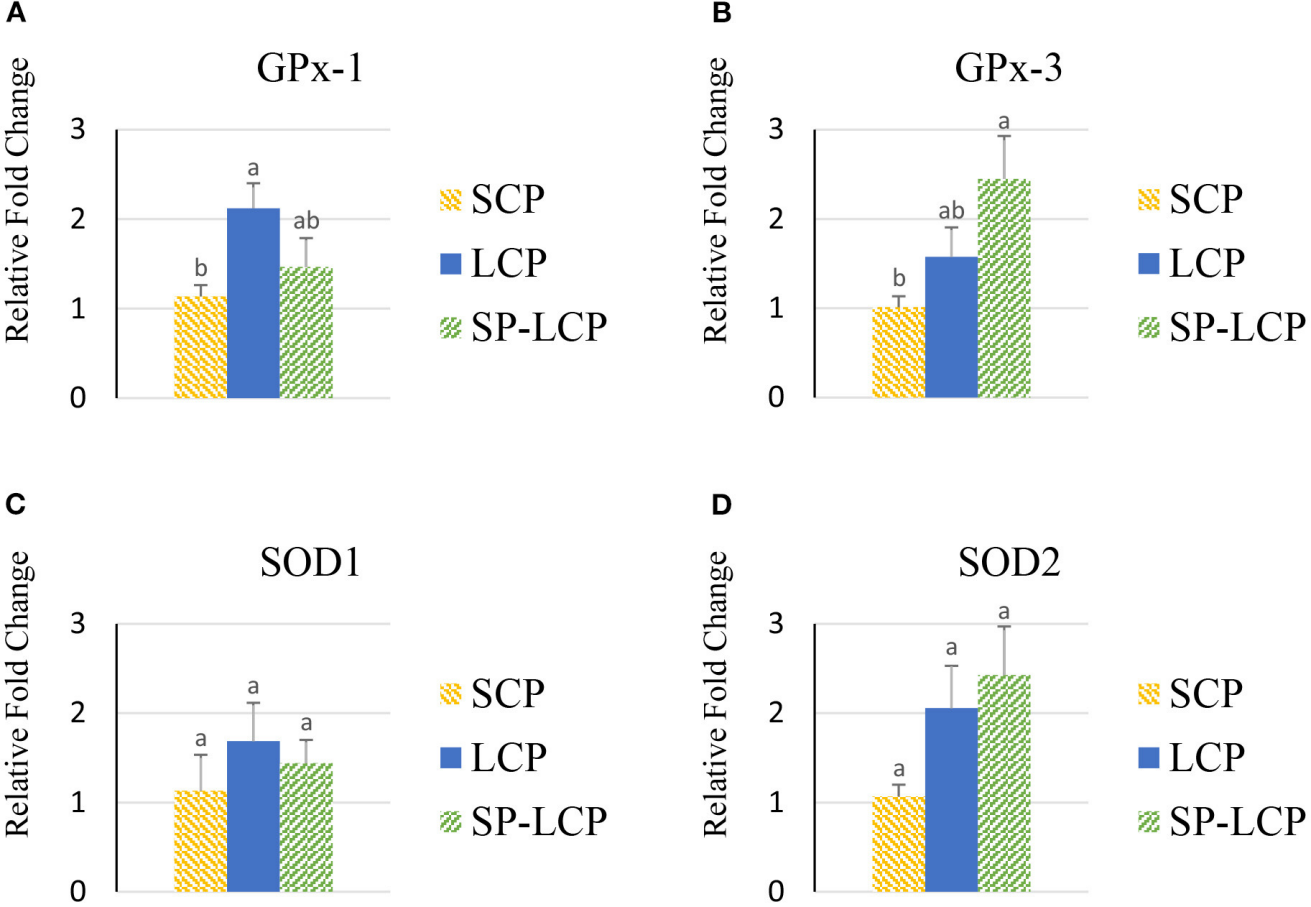
Effects of *Spirulina* on the expression of circulating antioxidant defense system. Relative mRNA abundance of glutathione peroxidase 1 (GPx-1) **(A)**, glutathione peroxidase 3 (GPx-3) **(B)**, superoxide dismutase 1 (SOD1) **(C)**, and superoxide dismutase 2 (SOD2) **(D)** in the blood of broilers on day (d) 37 were determined by real-time qPCR. Treatments include standard corn/soy (SCP) diet as control and low crude protein without (LCP) or with *Spirulina* (SP-LCP). Data are presented as mean ± SEM (*n* = 8/group). Means with different letters are significantly different (*P* < 0.05).

The LCP diet upregulated the blood expression of glutathione peroxidase 1 GPx-1 but not that of GPx-3 or superoxide dismutase 1 and 2 (SOD-1/2) compared with the control (SCP) group (Figures 6A–D). The inclusion of *Spirulina* reduced GPx-1 mRNA abundance to the control level; however, it non-significantly increased further the expression of GPx-3 and SOD-2 genes (Figures 6A,B,D).

## Discussion

Inflammation is a response to stress and can occur in multiple levels of severity, from immediate acute responses to long-term chronic conditions. There is an intertwining relationship between inflammatory response and oxidative stress, in which inflammation elevates free radicals to levels exceeding cell threshold, thus inducing oxidative stress (26). Stress-induced inflammation can be associated with a plethora of factors, including dietary nutrient composition, bacterial or viral infection, environmental factors, obesity, aging, and numerous diseases (27–33).

There is limited information on how low CP diets and *Spirulina* impact inflammatory biomarkers in broiler chickens. It is important to remember that “low” or “high” CP level is relative regarding species, age, environment, and other factors. Excessive dietary protein intake can cause undigested protein or amino acids to enter the hindgut, increasing the amounts of opportunistic bacteria such as *Clostridium perfringens* in the ileum and ceca of broilers (34). Excess dietary nutrients/metabolites could cause metabolic (meta)-inflammation, which is different from pathological and physiological inflammation, and typically occurs in modern broilers production systems due to several factors such as ingredients used in the diet, nutrient excess in the diet, and high feed intake (35). All forms of inflammation lead to an upregulation of certain inflammatory biomarkers (36). However, the mechanisms mentioned earlier could not be the case in our study, as, surprisingly, the LCP diet led to an increase in basophil concentration and inflammation-associated biomarkers.

Basophil concentrations vary widely between avian species but are more highly concentrated in birds than their mammalian counterparts (37). Basophils are highly granular leukocytes that are chemo-attracted to inflammation sites and undergo degranulation to release histamine and Th2 cytokines and increase expression of toll-like receptors (38). The significant increase in basophil counts in our study indicates a systemic inflammation that might be a result of increased bacterial translocation that was observed in the LCP-fed birds. This is supported by an increase in the expression of inflammatory cytokines and chemokines, including IL-3, IL-4, IL-6, IL-10, CCL-20, and TNF-α in response to the LCP diet. The increase in liver bacterial translocation might be the underlying cause of the observed inflammatory stress. Reducing dietary CP can increase the broiler's intestinal permeability and leakage, which in turn induces bacterial translocation (39–41). Bacterial translocation increases pro-inflammatory cytokine production (42), which adversely impacts growth performance, as nutrients are directed toward immune responses rather than growth (43). This is evidenced in our experimental conditions by lower bodyweight, bodyweight gain, and a 15-point increase in feed conversion ratio in LCP-fed birds compared with their SCP counterparts in unpublished data.

It is worth noting that Kamely et al. (44) reported no differences in inflammatory cytokine TNF-α and IL-1β expression in the abdominal cavity exudate of Ross 708 broilers. The discrepancy between the present study and Kamely et al. (44) suggests that a low protein diet might affect the inflammatory system in a tissue-specific manner [systemic in our experimental conditions vs. local or abdominal cavity in Kamely et al. (44)]. The difference in the experimental approaches (diet formulation, amino acid levels, etc.) might also contribute to the discrepancy mentioned earlier.

Supplementation of *Spirulina* to LCP diets alleviated these negative effects and reduced inflammation as evidenced by amounts of IL-3, IL-4, IL6, IL-10, CCL-20, C3, and NLRP3 mRNA that were not different from those in the SCP control diet. This was in conjunction with a seven-point increase in feed conversion ratio in algae-LCP fed birds compared with LCP in unpublished data from this trial. Qureshi et al. (19) noted that *Spirulina* enhances the immune response in chickens fed a standard CP diet, and numerous trials have shown that algae ameliorate the expression of inflammatory molecular signatures in other species (45–47).

Inflammasome-forming NLRP3 and non-inflammasome-forming NLRs (NLRC3, NLRC5, and NLRX1) help mediate inflammatory responses (48), such as the one witnessed in the LCP-fed birds. The NLRs are activated in response to pathogen-associated molecular patterns and damage-associated molecular patterns; however, the exact underlying mechanisms for many NLRs in chicken have yet to be elucidated. In mammals, for instance, it is known that following microbial stimuli, NLR detects molecular patterns in the cytosol and activates the NLRP3 inflammasome, which in turn activates caspase 1, resulting in the production of IL-18 and IL-1β (49). Chen et al. (50) suggested that NLRP3 inflammasome activation occurred *via* the TLR2-MyD88-nuclear factor-kappa B (NF-κB) signaling pathway when chickens were challenged with *Mycoplasma gallisepticum*. The upregulation of MyD88 expression in this study supports the involvement of the previously described pathway. MyD88 acts as a downstream signaling adaptor for all TLRs except TLR-3 (51). MyD88 binds to TLRs and IL-1 receptor families to activate mitogen-activated protein kinases, activator protein 1, and NF-κB pathways (52). NF-κB plays an important role in producing pro-inflammatory cytokines after stress induced by gram-negative bacteria infection, reactive oxygen species, oxidized low-density lipoprotein, and multiple other factors (53).

Previous studies in mammals described several additional secondary signals, including plasma membrane disruption for bacterial toxins, lysosome destabilization, and liposome-triggering mitochondrial reactive oxygen species (ROS), leading to inflammasome oligomerization and activation (54–56). Although the mechanisms by which *Spirulina* reduces bacterial translocation and reverses the pro-inflammatory state induced by LCP are not known at this time point, it is conceivable that *Spirulina* might enhance gut integrity and merits further in-depth investigations. In addition to enhanced intestinal barrier integrity, *Spirulina* contains high levels (180 mg/g) of the biliprotein *phycocyanin*, which has been shown to have antioxidant, antibacterial, and anti-inflammation properties (57–60). Hao et al. (61) recently described a regulatory mechanism by which phycocyanin inhibits NF-κB expression and inflammation in human lung cancer cells through downregulating toll/IL-1 receptor domain-containing adaptor protein. Park et al. (17), on the other hand, showed that *Spirulina* modulates the antioxidant system in broilers. In our experimental conditions, *Spirulina* was also shown to modulate the systemic antioxidant defense system by lowering GPx-1 and upregulating GPx-3 and SOD-2, which is quite intriguing. GPx catalyzes the reduction of various hydroperoxides to H_2_O *via* oxidation of reduced GSH into its disulfide form (62). SOD2, also known as mitochondrial manganese-dependent SOD, transforms toxic superoxide (a byproduct of the mitochondrial ETC) into hydrogen peroxide and diatomic oxygen (62). The differential expression between GPx-1 and GPx-3 suggests a potential compensatory mechanism; in other words, GPx-3 expression was upregulated by *Spirulina* to compensate for the lack of GPx-1, which might be depleted due to elevations in ROS production. The upregulation of SOD2 could result from increased xanthophyll content or perhaps suggests enhanced cytoprotection and clearance of mitochondrial ROS by *C-phycocyanin* derived from *Spirulina* (63), which might explain at least partly the reduction of inflammation and cytokine expression. *Spirulina* also has the xanthophyll lutein, which can increase SOD2 irrespective of ROS levels (64). In support of our hypothesis, *phycocyanin* is considered a principal component responsible for antioxidant activity in algae by scavenging hydroxyl radicals (65). Furthermore, carotenoids such as β-carotene also protect cells from oxidative stress through quenching singlet oxygen damage (66).

In conclusion, this is the first report, to our knowledge, defining potential molecular targets by which *Spirulina* inclusion reduces systemic inflammatory activity, including cytokines, chemokines, bacterial translocation, and proportions of basophils in broilers fed a low protein diet.

## Data Availability Statement

The original contributions presented in the study are included in the article/supplementary material, further inquiries can be directed to the corresponding author/s.

## Ethics Statement

The animal study was reviewed and approved by the University of Arkansas Animal Care and Use Committee (#21002).

## Author Contributions

GM, MK, WB, and SD: conceptualization. GM, GT-I, MK, and SD: methodology. GM, NE, and GT-I: software. GM, EG, NE, and SD: validation. GM, EG, GT-I, and GE: formal analysis. GM, EG, GT-I, MK, and SD: investigation. WB, MK, GT-I, GE, and SD: resources. GM, NE, and SD: data curation. GM: writing—original draft preparation. NE and SD: writing—review and editing. SD: visualization and supervision. WB, MK, and SD: project administration. WB, SD, and MK: funding acquisition. All authors contributed to the article and approved the submitted version.

## Conflict of Interest

The authors declare that the research was conducted in the absence of any commercial or financial relationships that could be construed as a potential conflict of interest.
